# Comparisons of Macro-Kinematic Strategies During the Rounds of a Cross-Country Skiing Sprint Competition in Classic Technique

**DOI:** 10.3389/fspor.2020.546205

**Published:** 2021-01-28

**Authors:** Finn Marsland, Judith Mary Anson, Gordon Waddington, Hans-Christer Holmberg, Dale Wilson Chapman

**Affiliations:** ^1^UC Research Institute for Sport and Exercise, University of Canberra, Bruce, ACT, Australia; ^2^Australian Institute of Sport, Bruce, ACT, Australia; ^3^Swedish Winter Sport Research Centre, Mid Sweden University, Östersund, Sweden

**Keywords:** cycle lengths, cycle rates, pacing, inertial sensors, performance analysis

## Abstract

This study was designed to examine macro-kinematic parameters of six female cross-country skiers during the qualifying, semi-final and final rounds of a 1.1 km sprint competition in classical technique. During each round these skiers were monitored continuously with a single micro-sensor, and their cycle parameters and relative use of these two sub-techniques calculated. Within each round six sections of the course, during which all skiers employed either double pole (DP) or diagonal stride (DS) sub-technique, were chosen for additional analysis. The mean macro-kinematic cycle parameters and relative usage of sub-techniques over the full course did not differ significantly between rounds. On average 54% of the course was covered employing DP and 13% using DS, while 32% was covered utilizing a non-cyclical or irregular technique. With DP, the mean racing speed and cycle rate (CR) on the starting, middle and finishing sections of the course differed significantly, with no differences in mean cycle length (CL) between the last two sections. At the finish, higher DP speed was achieved by increasing CR. On the three hills, where all athletes utilized DS, mean racing speed and CL, but not mean CR, differed significantly. On these sections DS speed was increased by utilizing longer cycles. The individual skiers utilized a variety of macro-kinematic strategies during different rounds and on different sections of the course, depending on individual strengths, preferences and pacing strategies, as well as the course topography and tactical interactions with other skiers. Such collection of macro-kinematic data during competitions can help to identify an individual skier's strengths and weaknesses, guiding the testing of different cycle rates, and lengths on different terrains during training in order to optimize performance.

## Introduction

Sprint events in cross-country skiing were introduced by the International Ski Federation (FIS) at World Cup in 1996, World Championships in 2001, and at the Olympic Winter Games in 2002 (FIS, 2018). These short 2–4 min events consist of a qualification round followed by head-to-head finals. Though some cross-country skiers can compete successfully at the highest level in both sprint and traditional distance events, many focus predominantly on Sprints and tailor their training accordingly. Typically, sprinters tend to have higher body mass and maximal sprint velocities, lower yearly training volumes and a higher proportion of anaerobic training (Losnegard and Hallén, 2014; Sandbakk and Holmberg, 2014).

Useful insights into sprint performance and physiology have been obtained from laboratory investigations (Sandbakk et al., 2012), including simulated sprint competitions on treadmills (Stöggl et al., 2007; Andersson et al., 2016, 2017). Although rollerski treadmills can be programmed with variable gradient and velocity to match on-snow course profiles, such methodology cannot simulate the changes in direction or head-to-head tactical interactions between skiers that occur in both sprint and mass-start competitions. Moreover, only performances on-snow in true winter and racing conditions can fully reflect the demands associated with cross-country skiing competition.

Analyses from simulated and real sprint competitions in the field, both on-snow (Zory et al., 2005, 2009; Andersson et al., 2010; Sandbakk et al., 2011; Mikkola et al., 2013; Haugnes et al., 2019) and on rollerskis (Vesterinen et al., 2009; Mikkola et al., 2010), have also provided valuable insights into pacing, fatigue and kinematic aspects of performance. Of the sprint studies conducted on-snow, both Mikkola et al. (2013) and Zory et al. (2009) and their colleagues examined skiing speed, cycle lengths and rates in the DP sub-technique on short (≤20 m) sections during simulated time-trials with no head-to-head racing, while Haugnes et al. (2019) contrasted these cycle kinematics of classical and freestyle skiing over the final 80 m of simulated sprint time trials. In order to extract macro-kinematic data and information on sub-technique usage over longer distances, Andersson et al. (2010) tracked skiers over an entire freestyle sprint time trial, using a snow-mobile and continuous video and GPS data collection. Of considerable interest in this context are recent developments in micro-sensor technology that enable continuous monitoring of velocity, cycle rates, cycle lengths and the extent to which the various sub-techniques are used during both training and competition (Myklebust et al., 2011; Marsland et al., 2012, 2015, 2017; Stöggl et al., 2014; Sakurai et al., 2016; Solli et al., 2018, 2020).

To our knowledge only one other study has collected macro-kinematic data continuously throughout both the time-trial and final rounds of a cross-country skiing sprint competition (Marsland et al., 2018). That particular study focused on the differences in macro-kinematics between sprint and distance competitions collected over common terrain under the same conditions, but did not examine individual macro-kinematic differences between the various sprint rounds. Accordingly, the purpose of this investigation was to compare individual sub-technique selection and associated macro-kinematic parameters during three different rounds of a classic sprint competition, to determine whether different strategies were utilized and to identify potential practical implications.

## Materials and Methods

### Participants

Six female cross-country skiers (age 24.8 ± 4.4 years, body height 1.66 ± 0.06 m, body mass 56.7 ± 5.2 kg, FIS Sprint Points 83.9 ± 64.6) were recruited to participate in the study. The participants included two World Cup or World Championship medallists and four Winter Olympians. The project was ethically approved by the University of Canberra and the Australian Institute of Sport, and all athletes signed an informed consent form.

### Event Format and Conditions

Data collection took place on a 1.1 km race track with various uphill, downhill and flat sections, as part of a FIS international sprint competition in classical style. The event took place at the start of the FIS competition season. All race participants first completed a qualifying time-trial (SPQ), then the top 12 were seeded into two semi-finals (there being too few competitors for quarter-finals). In accordance with the International Ski Federation's “lucky loser” system, the top two finishers from each semi-final (SPS) progressed directly to an A-final, as well as the next two fastest skiers from either semi-final. The remaining skiers participated in a B-final, so that each skier was involved in a third round of racing (SPF). The break between the qualification and the finals was 90 min, while all finals were completed within 45 min.

The temperature during the competition was stable, snow temperature rising from −2 to −1° and air temperature from −2 to +2°. The track was machine prepared by an experienced snow groomer. In the time-trial the skiers started in order of their FIS Sprint ranking order, lowest points first to highest last. Skiers used their own ski equipment, with skis prepared by their personal coaches, and warmed up for the event using their personal routines.

Our skiers were classified F1–6 based on their mean overall race speed in the sprint qualifying round (SPQ). Skiers F1, F4, and F5 then competed head-to-head in the first semi-final, while F2, F3, and F6 competed in the second semi-final. Skiers F1 to F5 all advanced to the A-final, which F2 won. F6 was the only one of our skiers who competed in the B-Final, which she won.

### Equipment

Data was collected from each athlete using a single micro-sensor unit (MinimaxX^TM^ S4, Catapult Innovations, Melbourne, Australia) positioned in the center of the upper back using a thin harness underneath the race bib. The unit contained a triaxial accelerometer (100 Hz, 6 g), gyroscope (100 Hz, 1,000 d/s) and GPS device (Fastrax, 10 Hz). The equipment and positioning were the same as used for similar cross country skiing competition analysis (Marsland et al., 2017, 2018). The accelerometer and gyroscope components were calibrated prior to data collection using a cradle supplied by the micro-sensor manufacturer, following the procedure described by Harding et al. (2008).

### Technique Classification

Micro-sensor data was imported into data analysis software (Makesens V70.6, Appsen, Canberra, Australia) and classified into sub-techniques using a previously developed algorithm. This algorithm used the accelerometer and gyroscope signals (processed by a low-pass Butterworth filter with cut-off frequencies of 2.0 and 1.0 Hz, respectively) to identify key features of each sub-technique, and is described fully by Marsland et al. (2015). The sub-techniques were classified as double pole (DP), kick double pole (KDP), diagonal stride (DS), tucking (Tuck), turning (Turn), and miscellaneous (Misc), and checked for accuracy by a cross-country skiing coach experienced in examining micro-sensor data. Obvious algorithm misclassifications were corrected and when classification was unclear the movement was designated as Misc. Cycles for each cyclical sub-technique were defined as being from one pole plant to the next pole plant on the same side.

### Track Sections

**Figure 1:**
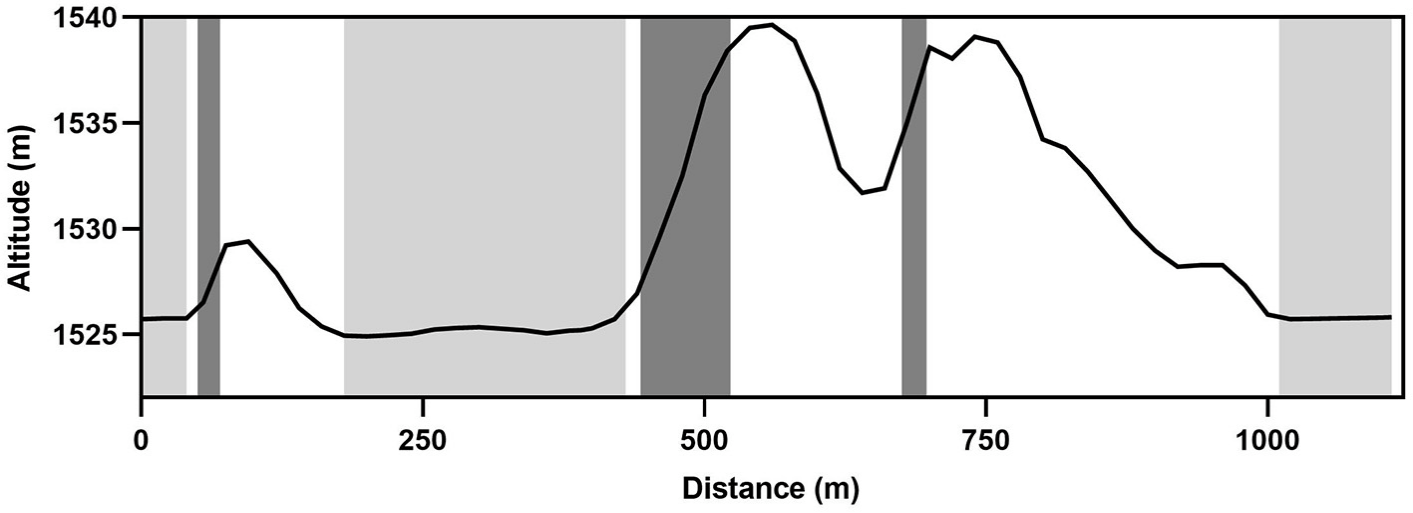
The topography of the sprint competition course including the location of six sections of the track used for within race comparisons. Light gray shading = double pole sections; dark gray shading = diagonal stride sections.

For comparison of cycle parameters during performance of DP and DS, three flat sections of the course and three hills were selected, respectively. The flat sections included the start, a middle section after a downhill ~180 m into the race, and the final straight section which was also after a downhill. The three hills, with average inclines of 13.5, 14.5, and 16.5%, began ~50, 440, and 680 m from the beginning of the course. The topography of the course and the individual sections are illustrated in Figure 1. The lengths of track used for DP analysis were ~43 m (Start), 252 m (Mid), and 104 m (Finish), with DS sections were 21 m (1st Hill), 80 m (2nd Hill), and 22 m (3rd Hill) long.

### Statistical Analysis

The values presented are means ± standard deviations (SD). The Wilcoxon matched-pair non-parametric test was used to compare the mean macro-kinematic parameters associated with the different sprint rounds and track sections, with an alpha level of *p* = 0.1 being considered significant. Statistical tests were performed in the GraphPad Prism 9 (GraphPad Software, San Diego, CA, USA) and Office Excel 365 (Microsoft Corporation, Redmond, WA, USA) software.

## Results

### Competition Outcomes and Overall Macro-Kinematics

**Figure 2:**
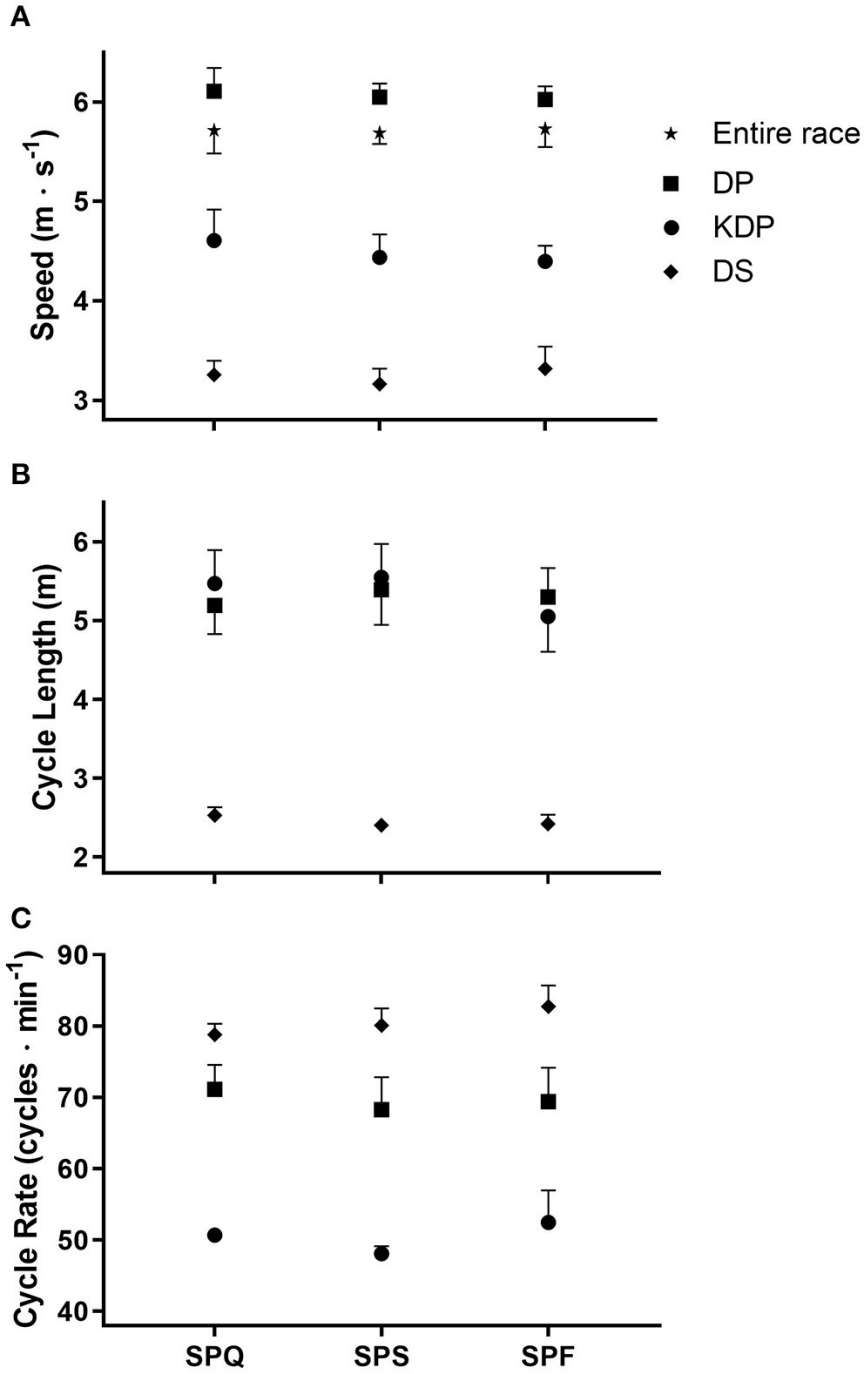
Mean racing speeds **(A)**, cycle lengths **(B)** and cycle rates **(C)** (± SD) for each of the three rounds of the cross-country skiing sprint competition. SPQ = Qualification round; SPS = Semi-finals; SPF = Final A or B; DP = double poling; KDP = kick double poling; DS = diagonal stride.

The six skiers had an overall mean race speed (S_RACE_) for all three sprint rounds of 5.7 ± 0.2 m·s^−1^, with overall mean sub-technique speeds S_DP_, S_KDP_, and S_DS_ of 6.1 ± 0.2, 4.5 ± 0.2, and 3.2 ± 0.2 m·s^−1^ respectively. The mean finishing time for all three rounds was 195.9 ± 6.4 s. No significant differences in any of these parameters during the three rounds were observed (Figure 2A). Moreover, the mean cycle lengths (CL_DP_ = 5.3 ± 0.4 m, CL_KDP_ = 5.3 ± 0.4 m, CL_DS_ = 2.5 ± 0.1 m) and cycle rates (CR_DP_ = 69.6 ± 4.2 cycles·min^−1^, CR_KDP_ = 50.7 ± 3.1 cycles·min^−1^, CR_DS_ = 80.6 ± 2.8 cycles·min^−1^) with the different sub-techniques did not differ between rounds (Figures 2B,C).

It should be noted that three skiers did not use KDP at any time, while one skier used this sub-technique in two rounds only. DP, used predominantly on flat and slight uphill sections, was associated with the fastest mean race speed and longest mean CL; while DS, utilized on steeper hills, was slowest with shortest mean CL. Notwithstanding the low use, KDP always has the lowest mean CR for classic sub-techniques due to each cycle featuring separated kicking and poling propulsion components.

DP was employed over 54 ± 3% of the distance covered during all three rounds, followed by Tuck (14 ± 2%), DS (13 ± 2%), Turn (8 ± 1%), and KDP (1 ± 1%), in that order. The remaining 10 ± 3% of Misc locomotion consisted largely of transitions between sub-techniques (3 ± 1%) and irregular techniques immediately before and after corners (4 ± 2%). Neither the percentage distance covered by nor the percentage time spent utilizing each sub-technique differed between rounds (Table 1). On average the skiers changed sub-technique 16 ± 2 times per round, or 14 transitions per kilometer.

**Table 1:** Mean sub-technique usage (% of distance and time ± SD) of our six skiers during the three rounds of the cross-country skiing sprint competition.

	**% of the total distance covered** **(% of the total time elapsed)**		
**Sub-technique**	**SPQ**	**SPS**	**SPF**
DP	54.2 ± 2.8 (51.2 ± 3.3)	53.5 ± 2.9(50.4 ± 2.9)	55.3 ± 2.9 (52.8 ± 3.3)
KDP	0.9 ± 2.1 (1.2 ± 2.7)	0.5 ± 0.9(0.7 ± 1.1)	0.3 ± 0.4 (0.4 ± 0.5)
DS	12.6 ± 1.5 (22.1 ± 2.0)	12.6 ± 1.1(22.6 ± 1.7)	11.8 ± 1.9 (20.4 ± 3.5)
Tuck	14.0 ± 2.6 (8.8 ± 1.9)	14.7 ± 1.5(9.1 ± 1.0)	13.2 ± 2.9 (8.3 ± 2.0)
Turn	8.1 ± 0.5 (5.9 ± 0.3)	8.3 ± 1.1(6.1 ± 0.9)	8.5 ± 0.7 (6.3 ± 0.5)
Misc	10.1 ± 3.6 (10.3 ± 2.8)	10.3 ± 2.5(10.5 ± 2.4)	10.9 ± 2.0 (11.3 ± 2.4)

*SPQ = Qualification round; SPS = Semi-finals; SPF = Final A or B; DP = double poling; KDP = kick double poling; DS = diagonal stride; Tuck = tucking; Turn = turning; Misc = all other techniques*.

### Section Analysis

**Figure 3:**
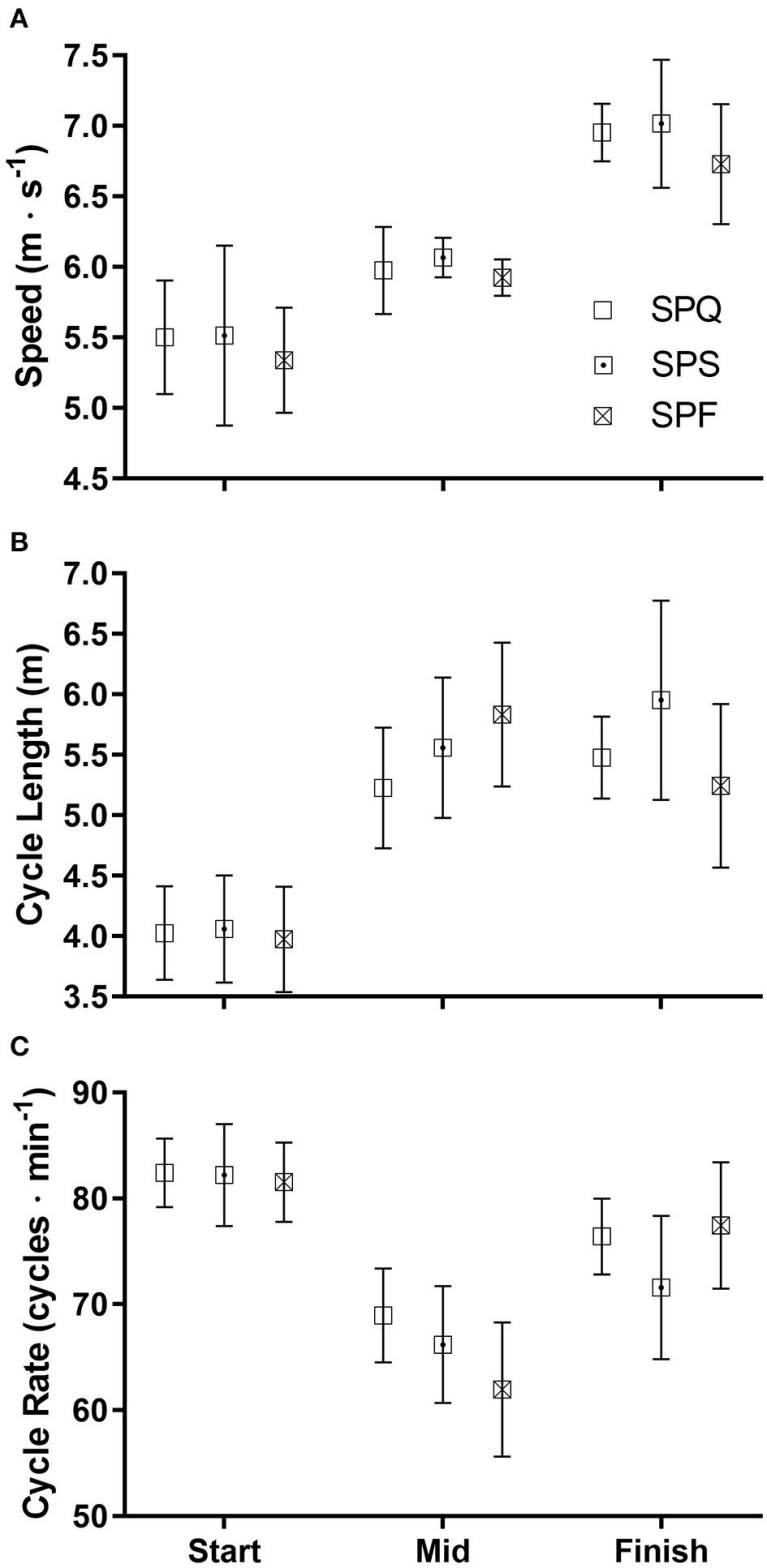
The mean double pole race speeds **(A)**, cycle lengths **(B)** and cycle rates **(C)** (± SD) during three sections of the course for each of the three rounds of the cross-country skiing sprint competition. SPQ = Qualification round; SPS = Semi-finals; SPF = Final A or B.

**Figure 4:**
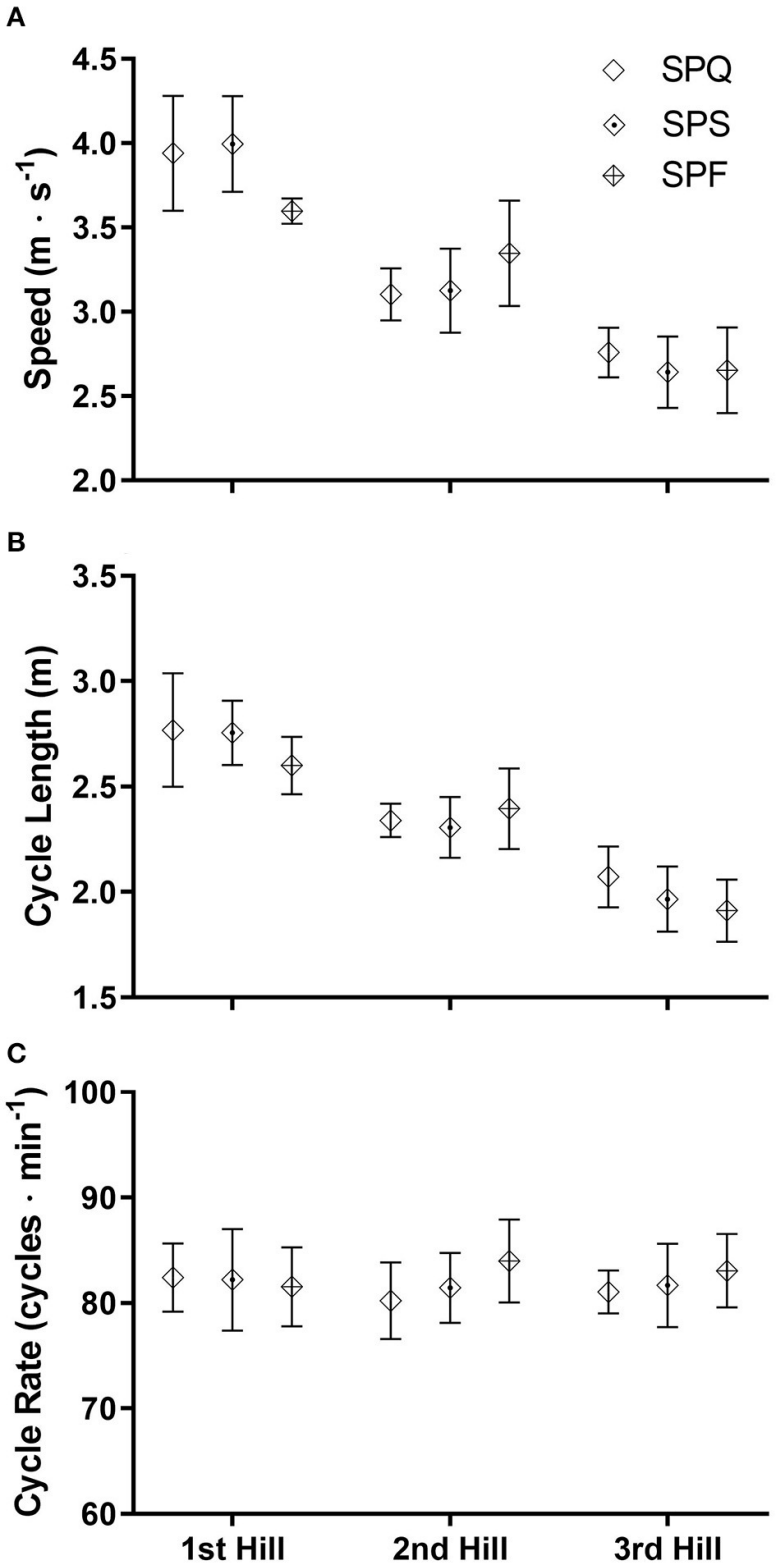
The mean diagonal stride race speeds **(A)**, cycle lengths **(B)** and cycle rates **(C)** (± SD) during three sections of the course for each of the three rounds of the cross-country skiing sprint competition. SPQ = Qualification round; SPS = Semi-finals; SPF = Final A or B.

The characteristics of the cycles analyzed are shown in Figures 3, 4. A small amount of locomotion in each section was classified as Misc (5.0 ± 4.0 m), including a fall by skier F1 in the final straight of the A-final. Across all three rounds the mean DP speed was slowest in the Start (5.5 ± 0.5 m·s^−1^), with a moderate speed in the Mid section (6.0 ± 0.2 m·s^−1^) and highest speed in the Finish straight (6.9 ± 0.4 m·s^−1^) (Figure 3A). CL was significantly lower during the Start (4.0 ± 0.4 m) compared to the other two sections, with no significant difference in CL between the Mid (5.5 ± 0.6 m) and Finish (5.6 ± 0.4 m·s^−1^,) sections (Figure 3B). Skiers consistently used the most rapid DP CR during the Start section (82.4 ± 3.6 cycles·min^−1^), with CR also high in the Finish (75.1 ± 5.9 cycles·min^−1^), with a significantly lower CR during the Mid section (65.7 ± 6.0 cycles·min^−1^) (Figure 3C).

There were no significant differences in DP race speed between rounds for each section, although in the SPF there was a slight trend toward a lower DP speed observed in the Finish straight. Within the Mid section there was a non-significant trend toward longer CL and less rapid CR as the event progressed through the finals, but this trend was not present in the Start or the Finish sections.

With DS, there were no notable differences in mean race speed, CL and CR in each section between rounds (Figures 4A–C). The mean CR also did not differ across each of the three hills (1st 85.9 ± 3.6, 2nd 81.9 ± 3.8, 3rd 81.9 ± 3.2 cycles·min^−1^). Speed and CL were highest on the first hill (3.8 ± 0.3 m·s^−1^, 2.7 ± 0.2 m), slower and shorter on the second hill (3.2 ± 0.3 m·s^−1^, 2.3 ± 0.1 m), and lowest on the third hill (2.7 ± 0.2 m·s^−1^, 2.0 ± 0.2 m). Thus, longer CL were used to achieve the increased DS speed.

### Individual Macro-Kinematics (Entire Course)

The three fastest skiers in the SPQ all had lower S_RACE_ (−2.5 ± 0.3%) and S_DP_ (−3.8 ± 2.8%) in the SPS; whereas the three slowest skiers during the SPQ were faster during the SPS (S_RACE_: +2.9 ± 0.7%; S_DP:_ −1.7 ± 1.9%) (Table 2). All skiers in the A-final (F1–5) demonstrated a higher S_DS_ in the SPF than the SPS (+6.3 ± 3.0%), with four of these five achieving this by enhancing their utilization of CR_DS_. The range of velocities during the SPS was more limited than during the SPQ or SPF.

**Table 2:** Mean overall race speed and speed, cycle length and cycle rate with the different sub-techniques our six skiers during each round ofthe cross-country skiing sprint competition. The values are presented as means ± SD.

		**Speed (m·s**^****−1****^**)**				**Cycle length (m)**			**Cycle rate (cycles·min**^****−1****^**)**		
**Skier**	**Round**	**Race**	**DP**	**KDP**	**DS**	**DP**	**KDP**	**DS**	**DP**	**KDP**	**DS**
F1	SPQ	6.0	6.4 ± 0.8	4.8 ± 0.2	3.4 ± 0.6	5.8 ± 0.9	5.7 ± 0.3	2.6 ± 0.7	66 ± 6	51 ± 2	80 ± 10
	SPS	5.8	6.2 ± 0.9	4.3 ± 0.3	3.1 ± 0.6	6.1 ± 1.1	5.3 ± 0.6	2.4 ± 0.6	62 ± 7	49 ± 2	80 ± 9
	SPF	5.8	6.0 ± 0.9	4.2 ± 0.0	3.4 ± 0.6	5.8 ± 1.1	4.8 ± 0.1	2.5 ± 0.5	64 ± 10	53 ± 1	83 ± 6
F2	SPQ	5.9	6.3 ± 0.9	–	3.5 ± 0.7	5.4 ± 0.8	–	2.7 ± 0.8	71 ± 7	–	80 ± 12
	SPS	5.8	5.9 ± 1.0	–	3.2 ± 0.6	5.6 ± 1.0	–	2.5 ± 0.8	64 ± 8	–	80 ± 11
	SPF	5.9	6.1 ± 1.1	–	3.4 ± 0.6	5.2 ± 1.0	–	2.4 ± 0.5	71 ± 10	–	87 ± 7
F3	SPQ	5.9	6.2 ± 0.9	–	3.1 ± 0.5	4.9 ± 0.8	–	2.5 ± 0.6	75 ± 5	–	78 ± 8
	SPS	5.7	6.0 ± 1.1	–	3.3 ± 0.6	4.9 ± 0.9	–	2.4 ± 0.5	73 ± 7	–	83 ± 6
	SPF	5.9	6.1 ± 1.1	–	3.6 ± 0.5	5.0 ± 0.9	–	2.6 ± 0.5	74 ± 10	–	83 ± 6
F4	SPQ	5.6	6.1 ± 1.0	4.7 ± 0.3	3.2 ± 0.6	5.0 ± 0.9	5.8 ± 0.5	2.4 ± 0.6	74 ± 6	50 ± 2	81 ± 9
	SPS	5.7	6.2 ± 1.1	4.6 ± 0.5	3.3 ± 0.6	5.4 ± 1.1	5.8 ± 0.5	2.4 ± 0.6	70 ± 6	47 ± 6	82 ± 9
	SPF	5.8	6.2 ± 1.2	4.4^*^	3.4 ± 0.5	5.4 ± 1.1	5.6^*^	2.4 ± 0.5	69 ± 8	48^*^	84 ± 8
F5	SPQ	5.5	5.8 ± 0.9	–	3.2 ± 0.7	4.9 ± 0.9	–	2.5 ± 0.6	71 ± 7	–	78 ± 9
	SPS	5.7	6.0 ± 1.2	–	2.9 ± 0.8	5.4 ± 1.2	–	2.3 ± 0.7	68 ± 9	–	76 ± 9
	SPF	5.6	5.8 ± 1.2	–	3.0 ± 0.6	5.6 ± 1.2	–	2.4 ± 0.6	63 ± 8	–	78 ± 7
F6	SPQ	5.3	5.9 ± 0.9	4.3 ± 0.5	3.1 ± 0.6	5.2 ± 0.9	5.0 ± 0.7	2.5 ± 0.6	69 ± 5	51 ± 3	77 ± 9
	SPS	5.5	5.9 ± 0.9	–	3.2 ± 0.6	4.9 ± 0.9	–	2.4 ± 0.6	73 ± 5	–	79 ± 8
	SPF	5.4	5.9 ± 0.9	4.5^*^	3.1 ± 0.6	4.8^*^± 0.9	4.8 ^*^	2.3 ± 0.5	74 ± 6	57^*^	81 ± 8

*F1–6 = fastest to slowest skier in the SPQ. SPQ = Qualification round; SPS = Semi-finals; SPF = Final A or B; DP = double poling; KDP = kick double poling; DS = diagonal stride*.

* *Only one KDP cycle was classified for this round*.

Regarding cycle lengths and rates, five skiers had longer CL_DP_ (+5.5 ± 3.4%) and slower CR_DP_ (−5.9 ± 2.8%) in the SPS than SPQ, with the slowest skier in the SPQ (F6) using a more rapid CR (+5.3%) and shorter CL (−4.4%) to achieve the most pronounced increase in S_DP_ (+2.6%) (Table 2). In contrast, all of the skiers except F4 exhibited shorter CL_DS_ in the SPS than SPQ (−5.8 ± 2.6%), even though only three (F1, F3, and F6) had an increased CR_DS_. The relatively small extent to which KDP was employed did not allow any meaningful comparison of CL or CR between rounds.

### Individual Sub-technique Usage

Skiers F1–5 used DS to a lesser extent in the SPS (−8.3 ± 4.3%) than the SPQ, with four of these employing even less DS in the SPF (Table 3). With the exception of F1 (who fell in one section while performing DP), all of the skiers used DP more extensively in the SPF than SPS. The two skiers (F1 and F5) who used DP least during the SPF used Tuck most.

**Table 3:** Usage of sub-technique (% of distance covered) by our six skiers during each round of the cross-country skiing sprint competition.

		**Cyclical Sub-techniques (%)**			**Non Cyclical Sub-techniques (%)**			
**Skier**	**Round**	**DP**	**KDP**	**DS**	**Tuck**	**Turn**	**Transitions**	**Other Misc**
F1	SPQ	55.2	2.5	12.3	15.6	7.8	3.5	3.1
	SPS	57.8	0.9	11.7	14.6	6.8	3.1	5.2
	SPF	53.6	0.8	9.9	16.9	8.8	5.5	4.4
F2	SPQ	49.8	0.0	15.0	14.0	6.9	3.7	10.6
	SPS	53.5	0.0	13.0	16.8	8.9	2.0	5.8
	SPF	54.8	0.0	10.1	12.1	8.7	2.6	11.8
F3	SPQ	57.6	0.0	11.9	15.5	7.1	2.3	5.6
	SPS	53.9	0.0	11.3	13.9	7.5	4.5	9.0
	SPF	60.1	0.0	11.2	9.2	9.5	2.5	7.5
F4	SPQ	50.6	2.6	15.3	10.1	8.2	5.0	8.2
	SPS	52.2	2.1	13.4	13.9	9.5	4.9	4.1
	SPF	56.9	0.5	13.7	12.5	7.7	2.2	6.5
F5	SPQ	53.5	0.0	12.7	17.1	7.6	3.3	5.8
	SPS	49.1	0.0	12.0	16.2	9.6	3.6	9.6
	SPF	51.8	0.0	11.4	16.3	8.6	3.6	8.3
F6	SPQ	54.3	5.3	14.1	11.8	7.5	2.3	4.8
	SPS	54.6	0.0	14.1	13.1	7.9	3.9	6.3
	SPF	54.7	0.4	14.5	12.2	7.4	4.1	6.7

*F1–6 = fastest to slowest skier in the SPQ. SPQ = Sprint qualification round; SPS = Sprint semi-finals; SPF = Sprint A or B final; DP = double poling; KDP = kick double poling; DS = diagonal stride; Turn = turning techniques; Tuck = downhill techniques with arms tucked; Misc = miscellaneous, i.e., all other techniques*.

## Discussion

Ours is the first detailed comparison of the usage of different techniques and associated macro-kinematic parameters during different rounds of a cross-country skiing sprint competition in classical style. Although the mean macro-kinematic parameters of our six participants did not differ significantly between rounds, these did differ on sections within the race. Moreover, individual skiers utilized sub-techniques to different extents, exhibiting variations in racing speed, CL and CR with each sub-technique.

### Overall Usage of Sub-techniques

As expected, DP was the predominant sub-technique employed, consistent with other analyses over entire classical cross-country skiing race courses (Marsland et al., 2017, 2018). KDP was utilized only by some of the skiers, mainly to transition between DS and DP. One of the first questions coaches and athletes ask when first inspecting a new sprint course is whether the entire course can be covered by double pole without grip wax. However, to discourage this tactic, the FIS has gradually changed course specifications and introduced new rules (FIS, 2016).

An interesting finding here was the 32.5 ± 3.3% of the total race distance covered utilizing the non- or semi-cyclical techniques Tuck, Turn and Misc (which includes transitions). Prior to recent studies involving continuous kinematic data collection technology (Marsland et al., 2017, 2018; Solli et al., 2018, Solli et al., 2020), observations concerning tucking have been limited to monitoring speed on short sections of track (Street and Gregory, 1994). Only a single recent study focused on the turning techniques of cross-country skiers (Bucher Sandbakk et al., 2014), although not during an actual competition. It is only through macro-kinematic full course analyses that the extent of Tuck and Turn usage in competitions have been quantified.

To a limited extent classification of these non- or semi-cyclical techniques by the algorithm applied to the micro-sensor data overlaps: if a skier had just stopped turning or had just stood up from tucking, but had not yet begun using a cyclical technique, this was classified as Misc; whereas if a skier was in a Tuck position while stepping to change direction, this was classified as a Turn. Similarly, Sakurai et al. (2016) found it difficult to distinguish between transitions and turns during skate rollerskiing on the basis of data provided by inertial sensors alone and therefore combined both of these into a single group. Nonetheless, it would be undesirable to ignore forms of skiing used to cover nearly 1/3 of the length of a competitive course, since improvements in these techniques are likely to improve overall performance. Indeed, such non-cyclical techniques should be included in training that simulates competition on real courses with curves and varied terrain, as well as subjected to technical analysis, e.g., with respect to the efficiency of transitions or downhill techniques.

### Comparison Between Sections

Characterization of cycle parameters while performing DP and DS sub-techniques on different sections of a race course can improve our understanding of both pacing and macro-kinematic strategies.

When accelerating out of the start all skiers used relatively shorter CL and more rapid CR to increase speed. When using DP on the flat section in the middle of the race, the skiers settled into a slower CR to conserve energy while still maintaining moderately high speed with long cycles. On the straight section into the finish, higher DP speeds were achieved using rapid CR while maintaining long cycles.

This observation that as skiers progress from sub-maximal to maximal DP speed, CL reaches a plateau while CR increases has also been reported in studies involving roller skiing (Lindinger et al., 2009) and skiing on snow (Nilsson et al., 2004). Similarly, in competition on snow, Haugnes et al. (2019) found that slower DP speed at the finish of a classic sprint race was correlated with a slower CR, with no significant change in CL.

The slow mean DP speed observed in the Start section in this study differs from other reports of higher DP speeds at the start than finish of a simulated classic sprint race (Vesterinen et al., 2009; Mikkola et al., 2013). However, in these other studies skiers were instructed to sprint maximally both at the start and finish of the race, whereas our skiers chose their own pacing strategies. In addition, these other investigators measured the start speed in a section 20 m from the start itself. Thus, the slower DP speed in the Start section observed here is likely to be due, at least in part, to the fact that the skiers were still accelerating, although the presence of a short steep hill only 50 m after the start may also have influenced pacing.

Interestingly, in the SPF the speed on the first hill tended to be slightly slower, as did the starting DP speed relative to the other rounds. This might reflect jostling by the athletes for position out of the start and their subsequent settling into a more tactical pace for skiing up the first hill.

In contrast to the range of mean CR employed in connection with performance of DP, the CR of our skiers was similar when performing DS on each of the hills, where changes in speed were achieved by varying CL. This finding is in contrast to those of Zory et al. (2005), who reported a correlation between CR and higher DS speed during a classical sprint world-cup competition on a 5% incline, with no increase in CL. However, it should be noted that in the present investigation the incline on the hills was not constant and closer examination of the data revealed shorter CL and more rapid CR on the steepest part of these hills. Such a correlation between increasing CR and gradient was also observed by Pellegrini et al. (2011) during DS rollerskiing on a treadmill and by Stöggl et al. (2018) during a classical distance cross-country skiing race. Unfortunately, the design of the present study did not allow precise examination of cycle characteristics relative to incline.

### The Macro-Kinematics of Individual Skiers Over the Entire Course

While performing each sub-technique, which our individual skiers utilized to different extents, they also used different cycle length and cycle rate. For example, among the three fastest skiers (F1–3) in the SPQ, F1 attained the fastest mean S_DP_ with the longest mean DP cycles and slowest mean CR_DP_, while F2 achieved almost the same S_DP_ with shorter and more rapid cycles. F3 reached a marginally slower S_DP_ using nearly the shortest CL_DP_ and highest CR_DP_, although this could be explained by her more extensive overall usage of DP, the fastest sub-technique, and less usage of DS. F3 evidently employed DP on steeper gradients, where more rapid and shorter cycles are required to maintain speed. Therefore, not unexpectedly, her mean CR_DP_ was faster and mean CL_DP_ shorter than those of the other skiers. This influence of topography on macro-kinematics has been noted previously (Marsland et al., 2018) and must be taken into consideration when interpreting mean data during an entire race.

Our skiers paced themselves as they progressed from the qualifying to the semi-final and then onto the final round of the competition. The more limited range of speeds in the SPS than the SPQ indicates that the slower skiers drafted and/or worked harder in order to keep up in the head-to-head racing, while the faster skiers may have been conserving energy for the final. In this connection distance runners are known to maintain a higher pace when running in a group than when running alone (Hanley, 2015). Moreover, Bilodeau et al. (1995) found that skiers can conserve energy using the classical technique by drafting behind other skiers, although not as much energy can be conserved in this manner as during road cycling, which involves higher speeds. In a more recent study of sprint cross country skiing in freestyle technique, Andersson et al. (2019) reported increased tactical effects on pacing and likely use of drafting during head-to-head rounds, however the higher racing speeds in the knock-out rounds witnessed in that study were not observed with our skiers.

In this context it can be asked whether our slower participants could have skied faster in the SPQ if they had chosen a different kinematic strategy. For example, could F6 have skied as rapidly in the SPQ as in the SPS by increasing her CR_DP_, or was the head-to-head effect alone responsible for enhancing her speed? It might be beneficial to use a more rapid CR_DP_ during high intensity training across different terrain, both individually and head-to-head with other skiers, to ensure that skiers who self-select a slower CR_DP_ are capable of maintaining a higher rate when racing.

During the SPF, the four fastest skiers (note that F1 would have had a higher S_RACE_ had she not fallen) were able to maintain or increase their speed, indicating that they had energy in reserve; whereas F5 and F6 could not achieve as high a mean speed as in the SPS, perhaps due to fatigue. However, F6 may have only skied as fast as was necessary to win the B-final. In the A-final, all five skiers demonstrated a higher cycle rate and velocity with the DS than during the SPS, with F3, in particular, increasing their cycle length as well.

This ability to adapt skiing speed and cycle rate during different rounds of a competition and/or on different sections of a race course appears to be of considerable importance. An interesting question in this context is whether slower and longer cycles in earlier rounds are associated with less fatigue and thereby allow faster cycles in the final round. Several of the authors have been told that skiers have used DS during earlier rounds of classic sprint events in order to conserve their upper body for an all-out DP effort in the final round.

### Individual Sub-technique Usage Over an Entire Course

The observation here that during successive rounds of a competition better skiers tend to use DS less-and-less and DP more-and-more on moderate inclines may have several explanations, including conscious choice of the faster technique more often when racing head-to-head and in the final round. Alternatively, increased tucking could indicate fatigue or something as simple as grip wax becoming less effective could cause skiers to switch from DS to DP earlier. Future research will determine whether this change in sub-technique usage is characteristic of our individual skiers or a general phenomenon. In the latter case, having energy reserves for powerful DP in the final round would appear to be advantageous, a situation that would influence both racing tactics (using the legs more to conserve the upper body) and training (using more DP later during simulated races).

### Limitations and Future Perspectives

The main limitation associated with interpretation of macro-kinematic characteristics over an entire course is the influence of topography on the usage of different sub-techniques. Although information concerning the relative usage of the different sub-techniques is in itself valuable, variations in speed, CL and CR throughout the race may make mean values misleading, especially since different skiers may utilize different sub-techniques on the same section (as noted earlier under *Macro-kinematic observations*).

Potentially we could also have analyzed continuous sections of the course without corners and downhills, thereby eliminating the need for the Tuck and Turn classifications and reducing the remaining Misc classifications to transitions and irregular technique (such as changing tracks, stumbling or falling).

Analysis of both entire races and of sections on which all skiers employ the same sub-technique can help training and choice of race techniques and strategies, and both should be included in future analyses.

## Conclusion

The macro-kinematic strategies utilized during cross-country skiing competition are complex. Not only can skiers vary their CL and CR to achieve optimal racing speed, they can also change sub-technique at will in response to varying terrain. In addition, in the case of sprint races, optimal strategies involve not only employing a combination of sub-techniques to ski rapidly enough to progress to the next round, but also conservation of as much energy as possible in order to be more competitive in the final round.

Here, we demonstrated how cycle characteristics during a classical sprint race are influenced by both the topography of the course and individual tactics. We also show how continuous collection of macro-kinematic data on an individual skier might assist in identifying his/her strengths and weaknesses, thereby helping to optimize training and refine racing strategies. Other potential practical recommendations include employing different combinations of sub-technique cycle rates and lengths over varied terrain, as well as taking course topography into consideration when deciding on a macro-kinematic racing strategy. Future monitoring of more of the world's best skiers while they race on sprint courses with different profiles should help identify optimal macro-kinematic strategies.

## Data Availability Statement

The dataset presented in this article may be obtained by contacting the corresponding author finn.marsland@gmail.com.

## Ethics Statement

The studies involving human participants were reviewed and approved by University of Canberra Human Ethics Committee. The patients/participants provided their written informed consent to participate in this study.

## Author Contributions

FM collected and analyzed the data, prepared the results, and wrote the first draft of the manuscript. All authors contributed to the study design, interpretation of results, and the manuscript revision.

## Conflict of Interest

The authors declare that the research was conducted in the absence of any commercial or financial relationships that could be construed as a potential conflict of interest.
